# A probiotic has differential effects on allergic airway inflammation in A/J and C57BL/6 mice and is correlated with the gut microbiome

**DOI:** 10.1186/s40168-021-01081-2

**Published:** 2021-06-10

**Authors:** Mateus B. Casaro, Andrew M. Thomas, Eduardo Mendes, Claudio Fukumori, Willian R. Ribeiro, Fernando A. Oliveira, Amanda R. Crisma, Gilson M. Murata, Bruna Bizzarro, Anderson Sá-Nunes, Joao C. Setubal, Marcia P. A. Mayer, Flaviano S. Martins, Angélica T. Vieira, Ana T. F. B. Antiorio, Wothan Tavares-de-Lima, Niels O. S. Camara, Rui Curi, Emmanuel Dias-Neto, Caroline M. Ferreira

**Affiliations:** 1 Department of Pharmaceutics Sciences, Institute of Environmental, Chemistry and Pharmaceutical Sciences, Universidade Federal de São Paulo, R. São Nicolau, 210, Diadema, SP 09913-03 Brazil; 2 Department CIBIO, University of Trento, Trento, Italy; 3 Medical Genomics Laboratory, CIPE/A.C. Camargo Cancer Center, São Paulo, Brazil; 4 Department of Biochemistry, Institute of Chemistry, Universidade de São Paulo, São Paulo, Brazil; 5 Center for Mathematics, Computing and Cognition (CMCC), Federal University of ABC – UFABC, São Bernardo do Campo, SP Brazil; 6 Department of Clinical Analyses, Universidade Federal do Paraná, Curitiba, Brazil; 7 Department of Medical Clinic, Faculty of Medicine, University of São Paulo, São Paulo, 01246-903 Brazil; 8 Department of Immunology, Institute of Biomedical Sciences, Universidade de São Paulo, São Paulo, Brazil; 9 Department of Microbiology, Institute of Biomedical Sciences, University of São Paulo, São Paulo, SP Brazil; 10 Department of Microbiology, Institute of Biological Sciences, Federal Universidade de Minas Gerais, Belo Horizonte, Brazil; 11 Department of Biochemistry and Immunology, Biological Science Institute, Federal University of Minas Gerais, Belo Horizonte, Brazil; 12 Department of Pathology, School of Veterinary Medicine and Animal Science, Universidade de São Paulo, São Paulo, Brazil; 13 Department of Pharmacology, Institute of Biomedical Sciences I, Universidade de São Paulo, São Paulo, Brazil; 14 Interdisciplinary Post-Graduate Program in Health Sciences, Cruzeiro do Sul University, São Paulo, Brazil; 15 Laboratory of Neurosciences (LIM-27), Institute of Psychiatry, Medical School, Universidade de São Paulo, São Paulo, Brazil

**Keywords:** Gut microbiota, Probiotic, Experimental allergic disease, Airway

## Abstract

The phenotypes of allergic airway diseases are influenced by the interplay between host genetics and the gut microbiota, which may be modulated by probiotics. We investigated the probiotic effects on allergic inflammation in A/J and C57BL/6 mice. C57BL/6 mice had increased gut microbiota diversity compared to A/J mice at baseline**.** Acetate producer probiotics differentially modulated and altered the genus abundance of specific bacteria, such as *Akkermansia* and *Allistipes*, in mouse strains. We induced airway inflammation followed by probiotic treatment and found that only A/J mice exhibited decreased inflammation, and the beneficial effects of probiotics in A/J mice were partially due to acetate production. To understand the relevance of microbial composition colonization in the development of allergic diseases, we implanted female C57BL/6 mice with A/J embryos to naturally modulate the microbial composition of A/J mice, which increased gut microbiota diversity and reduced eosinophilic inflammation in A/J. These data demonstrate the central importance of microbiota to allergic phenotype severity.

Video Abstract

**Supplementary Information:**

The online version contains supplementary material available at 10.1186/s40168-021-01081-2.

## Introduction

Asthma is an airway inflammatory disease that exhibits quite heterogenous severity and treatment responsiveness, which are likely due to the diverse mechanisms underlying this disease. An established approach to asthma treatment involves the categorization of patients into so-called phenotypes, which are defined by observable characteristics that result from a combination of hereditary and environmental influences [1]. Patients may respond differently to the same therapeutic interventions despite similar clinical symptoms [2, 3].

Several groups used various approaches to identify genes that may be related to asthma development over the past two decades. Some researchers used different inbred strains of mice under the same controlled environments to estimate the influence of genetic background on the allergic airway inflammation phenotype [4–6]. These researchers observed that some mouse strains, such as A/J mice, showed greater eosinophilia after 24 h of a challenge with ovalbumin (OVA) than other mouse strains, such as C57BL/6 mice [7, 8]. Animal and clinical studies on the relevance of host genetics to asthma clearly indicated that gene-environment interactions were involved in the manifestations of this complex disease [9, 10]. An imbalance of the microbial communities in the gut and lung has been increasingly associated with the incidence and severity of asthma [11–14]. More accurate pathobiological mechanisms in asthmatic patients may be elucidated from further analyses of the composition and metabolic activity of an individual’s microbiota [15].

The gut microbiome may play a role in asthma, and strategies to modulate the microbiota, such as probiotic supplementation, should be investigated. However, there is no consensus on whether probiotic supplementation prevents or alleviates asthma symptoms. Clinical studies are impacted by heterogeneity, such as the different environments and dietary habits of participants, which may interfere with probiotic treatment and lead to different outcomes. Experimental studies generally use different probiotic species in different mouse strains and various protocols to induce allergic inflammation, which complicates relevant comparisons and validations. All of these factors create difficulty in obtaining reliable conclusions on the impact of the use of probiotics in asthma.

Approximately 3.9 million adults in the USA use prebiotic or probiotic supplements [16], and some studies reported probiotic-associated morbidity and mortality [17, 18]. Therefore, additional studies on this topic are necessary. Many asthma patients likely consume probiotics, and probiotics may play a role in disease control. Therefore, an understanding of probiotic microbiota-host interactions is truly relevant. We investigated the role of specific probiotics in the two different hosts, A/J and C57BL/6 mice, and the relevance of the gut microbiota composition in experimental allergic disease.

Understanding the relevance of probiotic-host interactions may call attention to the indiscriminate use of probiotic products by asthmatic consumers and reveal the possible benefits/risks of these products in asthma patients.

## Materials and methods

### Mouse experiments

Male A/J and C57BL/6 mice were obtained from the animal facility of the Institute of Biomedical Sciences (University of Sao Paulo, Brazil) but were originally from Jackson Laboratories. Mice were housed in specific pathogen-free conditions in the same animal room. All mice used were age-matched and fed a standard chow diet of irradiated AIN93-M [19]. Mice in the experiment described in Fig. S1 received a modified AIN93-M diet containing 15% citrus pectin (Vetec, Duque de Caxias, RJ, Brazil). Littermates of the same sex and age were randomly assigned to different experimental groups. For the experiment described in Fig. 4, one female was used.

### Study design

All mice were 5 weeks old when probiotic treatment was started. Acetate measurement and microbiome analysis were performed 1 day before OVA sensitization. The effects *Bifidobacterium longum* 5^1A^ were evaluated in an experimental model for lung inflammation using C57BL/6 and A/J mouse strains. Lung inflammation was induced via sensitization with ovalbumin (OVA). To assess whether the effect of the probiotic was due to acetate production, the effect of systemic acetate was also evaluated in OVA-induced inflammation. The probiotic or sodium acetate was given to the experimental groups, and controls only received vehicle. The animals were euthanized, and lung inflammatory parameters were determined. To investigate whether the results were due to the genetic background of the mouse strains or their microbiome, the same protocols for experimental lung inflammation and probiotic administration were performed in A/J mice born from C57BL/6 females.

### Induction of allergic lung inflammation

Mice were sensitized via the intraperitoneal (i.p.) injection of 30 μg and 50 μg of OVA grade V (Sigma Chemical Co., St. Louis, MO, USA), dissolved in 200 μl of sterile phosphate buffer saline (PBS) and 1.6 mg of inject alum (Prod# 77161, Thermo Scientific, Rockford, USA) on days 0 and 7, respectively. Mice were challenged with 10 μg of OVA in 20 μl of intranasal (i.n.) sterile PBS on day 14 and 20 μg of OVA in 30 μl of intratracheal (i.t.) sterile PBS on days 21 and 22. The animals were euthanized 24 h after the last challenge (i.e., day 23) [20]. Controls receiving saline were also tested.

### Acetate administration

Mice were treated with sodium acetate (Sigma-Aldrich, St. Louis, MO, USA) at 1 g per kg body weight diluted in sterile PBS via intraperitoneal injection starting15 days before exposure to OVA and every other day throughout the experimental period [21]. Controls receiving saline were also tested.

### Probiotic administration

*Bifidobacterium longum* 5^1A^ was obtained from the Laboratory of Biotherapeutic Agents, Department of Microbiology, Institute of Biological Sciences, Federal University of Minas Gerais. The bacterium was isolated in the city of Salvador (Bahia, Brazil) and identified using morphological, respiratory, and biochemical tests [22], followed by multiplex PCR [23]. The bacterium was cultivated in MRS broth (Difco) under anaerobic conditions in an anaerobic jar at 37 °C for 48 h. Aliquots of 100 μl *Bifidobacterium longum* 5^1A^ suspension in PBS (1 × 10^9^CFU/ml) were intragastrically administered using a gavage needle to mice daily, starting 15 days before the first sensitization and throughout the experimental period until the last challenge with OVA (day 22). Heat-killed *Bifidobacterium longum* 5^1A^ was prepared by heating aliquots of viable bacteria suspensions for 20 min at 80 °C and administered as described above for live bacteria. The control group received only the probiotic vehicle (PBS).

### Embryo transfer

Ovulation was induced in A/J female mice via the intraperitoneal administration of 5 IU PMSG between 12:00 and 2:00 p.m. Two days later, 5 IU of hCG was injected intraperitoneally after random mating with A/J male mice. At 0.5 days post-coitum (d.p.c.), female mice with a copulatory plug were separated from male mice. At 1.5 d.p.c., female A/J mice were euthanized via cervical dislocation. The abdominal cavity was opened, and the oviducts were aseptically collected. Embryos were flushed from the oviducts using M2 medium (supplemented with Pen-Strep 100x) and collected in a pool with a mouth transfer capillary setup under a stereoscopic microscope (SMZ-10, Nikon). Two-cell embryos with intact zona pellucida were washed 10 times in M2 medium. Recipients consisted of nulliparous C56BL/6 female mice. Each female was mated with a vasectomized male mice C57BL/6 near the end of the light cycle period. Embryo transfers were performed at 0.5 d.p.c. in recipient females with a copulatory plug. Surgeries were performed within the SPF facility clean area under a horizontal laminar flow cabinet. Females were anesthetized with 0.2% acepromazine (2 mg/kg), 10% ketamine hydrochloride (100 mg/kg), and 2% xylazine hydrochloride (10 mg/kg) via intraperitoneal injection. A sterile saline solution was used to prevent corneal drying during the surgery. Each female was placed in sternal recumbency on a digital heating plate at 37 °C. The peritoneal cavity was accessed via a musculature incision over the ovary fat pad. Under stereoscopic microscopy (SMZ 2-B, Nikon), a small incision was made in the bursa between the ovary and the oviduct to expose the infundibulum. Each female received 20 embryos equally that were divided between the oviducts using a glass micropipette. Pups were born in 3 weeks and weaned after 21 days.

### Bronchoalveolar lavage fluid (BALF)

The trachea was cannulated after euthanasia. The lungs were washed twice with 0.8-ml aliquots of PBS injected through the cannula. The total number of cells in BALF was counted using a Countess® Automated Cell Counter (Invitrogen). The BALF was centrifuged at 290*g* for 1 min at 4 °C, and the supernatant was collected and stored at − 80 °C for cytokine analysis. The cells were resuspended at 5.0 × 10^5^ cells/ml. Differential cell counts were performed using cytocentrifuge analysis and prepared from aliquots of BALF (200 μl) centrifuged at 45*g* for 1 min using a cytocentrifuge (Fanem, São Paulo, Brazil). Cells were stained with Instant Prov (Newprov, São Paulo, Brazil), and a total of 300 cells were counted to determine the proportion of neutrophils, eosinophils, and mononuclear cells using standard morphological criteria.

### Histological analyses

Histopathological analysis was performed on samples from the lungs of the OVA/saline, OVA/probiotic, and OVA/acetate mouse groups. To assess the pathological changes, the lungs were removed from mice after BALF collection and fixed via immersion in 4% paraformaldehyde. The lobes were sectioned sagittally, embedded in paraffin, cut into 5-μm sections, and stained with periodic acid-Schiff (PAS). Mucus production and lung inflammation were measured as previously described [24]. Goblet cell hyperplasia in the airway epithelium was evaluated using the following scoring system; a numerical score was determined for the abundance of PAS-stained cells in the tracheal section as follows: 0, < 5%; 1, 5–25%; 2, 25–50%, 3, 50–75%; and 4, > 75%. Two different people blindly analyzed the sections.

### Measurement of cytokine production

BALF samples from animals were collected and centrifuged at 1200 rpm for 5 min. The supernatants were stored at − 80 °C for cytokine analysis. Cytokine concentrations were assayed using interleukin (IL)-4 and interferon gamma (IFN-γ) ELISA kits (BD Biosciences) according to the manufacturer’s instructions. All determinations were performed in duplicate.

### Determination of OVA-specific IgE, IgG1, and IgG2a production

Plates were coated with 5 μg/ml of OVA solution in carbonate buffer (pH 9.5) at 4 °C overnight. Plates were washed with a wash buffer (PBS containing 0.05% of Tween-20) and blocked in assay buffer (PBS containing 10% heat-inactivated fetal bovine serum) for 1 h at room temperature (RT). Mouse serum samples were diluted in assay diluent in 1:1000 (v/v) and added to the plates followed by a 2-h incubation at RT. The plates were washed, and peroxidase-conjugated secondary antibodies were added for 1 h at RT: anti-IgG1-HRP (GeneTex, USA) diluted 1:20,000 (v/v) in assay diluent or anti-IgG2a-HRP (GeneTex, USA) diluted 1:5000 (v/v). The plates were washed, and TMB Substrate Reagent Set (BD Biosciences) was added and incubated for 30 min at RT in the dark. The reaction was stopped with the addition of 2 N sulfuric acid. Absorbance was acquired at 450 nm. The values were calculated after subtracting the absorbance of a well with no serum added (incubated only with assay diluent). The results are presented in optical density (O.D.) units, and the ratio was calculated by dividing the respective values, as modified from [25]. OVA-specific IgE concentrations were assayed using an ELISA kit (Cayman Chemical).

### EPO assay

The eosinophil peroxidase (EPO) assay was used to estimate eosinophil numbers in lung tissue [26, 27]. After flushing the pulmonary artery with 20 ml of PBS, the left lung was weighed, chopped, and homogenized in PBS (5% [wt/vol]) using a tissue homogenizer (PowerGen 125; Fisher Scientific, Pittsburgh, PA, USA). The homogenate was centrifuged (3000×*g* for 10 min), and the red blood cells in the pellet were lysed. Cells were resuspended in PBS (pH 7.4) containing 0.5% hexadecyltrimethylammonium bromide (Sigma-Aldrich). The cell solution was homogenized, and the homogenates were subjected to three rounds of freeze/thaw in liquid nitrogen. Homogenates were stored at − 20 °C until assayed. Samples of lung tissue were centrifuged, and the supernatant was diluted 1:3 in PBS/HTAB. The assay was performed in 96-well plates (Nalge Nunc International Co., Naperville, IL). Each sample was tested in triplicate with the addition of 75 μl of the sample/well, 75 μl of SIGMAFAST™ OPD substrate (Sigma-Aldrich), and 6.6 mM hydrogen peroxide in 75 mM Tris-HCl, pH 8.0/well. The reaction was performed at 20 °C for 30 min and was stopped with a 4-M sulfuric acid solution. Plates were read at 492 nm on a microplate reader (Titertek Multiskan), and the results are given in absorbance units.

### Lung function analysis

All animals were anesthetized with ketamine (100 mg/kg, i.p.) and xylazine (20 mg/kg) and paralyzed with pancuronium bromide, and a stable depth of anesthesia was maintained [28]. After tracheostomy, the trachea was cannulated using a blunt 18-gauge metal tube, and the mouse was ventilated using a computer-controlled small-animal ventilator (flexiVent; SCIREQ, Montreal, QC, Canada) and a tidal volume of 10 ml/kg and a respiratory frequency of 150 breaths/min. A positive end-expiratory pressure (PEEP) of 2 cm H_2_O was applied throughout. An external jugular vein was isolated for an intravenous (i.v.) infusion of methacholine (MCh). At the outset, 6 μg of MCh was provided intravenously to ensure that the animal was responsive to MCh and that airway resistance returned to the baseline value after the MCh-induced increase, which indicated that the mouse was in a stable physiological condition. To obtain a dose-response curve, a bolus of MCh was injected starting at a dose of 4 μg (200 μg/ml solution in PBS; i.v. boluses of 10–40 μl). Prior to each MCh dose, the expiratory path was obstructed for 15 s to produce a deep inflation, after which exhalation was immediately allowed. Ventilation was continued for approximately 2 min between consecutive MCh doses. Airway responsiveness was equal to the Newtonian resistance (Rn) [28].

### Flow cytometry

Mouse Treg cells were collected from the BALF and analyzed for CD4+ CD25+ Foxp3+ expression using a mouse Treg cell staining kit containing APC-labeled anti-CD4, PE-labeled anti-CD25, and FITC-labeled anti-Foxp3 (eBioscience) according to the manufacturer’s instructions. Briefly, prepared cells (1 × 10^6^) were washed via centrifugation with cold PBS, resuspended in 1 ml of fixation/permeabilization solution, and incubated in the dark at 4 °C for 30–60 min. The cells were washed once with 2 ml of permeabilization buffer, collected via centrifugation, resuspended in 20 ml of blocking agent with 2 ml of 2% normal rat serum in permeabilization buffer, and incubated at 4 °C for 15 min. A fluorochrome-conjugated antibody or isotype control in 20-ml permeabilization buffer was added, followed by incubation in the dark at 4 °C for 30 min. The cells were washed with 2 ml of permeabilization buffer, resuspended in flow cytometry buffer (PBS with 2% FBS), and analyzed using a FACSCanto II cytometer (BD Bioscience, San Diego, CA, United States). The data were analyzed using the FlowJo® software.

### SCFAs measurement

To measure short-chain fatty acids (SCFAs) in the serum, 20 mg of sodium chloride, 10 mg of citric acid, 20 μl of 1 M hydrochloric acid, and 100 μl of butanol were added to 100 μl of serum samples. To quantify SCFAs, a calibration curve for the concentration range of 0.015–1 mg/ml was constructed. SCFA measurements were performed following a recently published protocol [29]. Chromatographic analyses were performed using an Agilent 6850 system with the ExChrom software, equipped with a 7683B automatic liquid sampler, a flame ionization detector (FID) (Agilent Technologies, USA), and a fused-silica capillary RTX WAX (Restec Corporation, USA) with dimensions of 60 mm × 0.25 mm internal diameter (i.d.) coated with a 0.15-μm-thick layer of polyethylene glycol. The initial oven temperature was 100 °C (hold 2 min), which was increased to 200 °C at a rate of 15 °C/min (hold 5 min). The FID temperature was maintained at 260 °C, and the flow rates of H_2_, air, and the make-up gas N_2_ were 35, 350, and 25 ml/min, respectively. Sample volumes of 1 μl were injected at 260 °C using a split ratio of approximately 25:1. Nitrogen was used as the carrier gas at 25 ml/min. The runtime for each analysis was 12.95 min.

### Microbial community profiling

Fecal samples were collected 1 day before the induction of experimental lung inflammation. Fecal pellets were collected in a laminar flow hood and frozen in 2-ml tubes at − 80 °C until DNA extraction. Pellets were placed in MoBio PowerSoil bead tubes and incubated at 70 °C for 10 min before proceeding with the manufacturer’s recommended protocol. The V4–V5 region of the 16S rRNA gene was amplified using the forward 5′-AYTGGGYDTAAAGNG-3′ and reverse primer 5′-CCGTCAATTCNTTTRAGTTT-3′. Three 20-μl amplification reactions were performed per sample, each containing 2.5 μM of each primer, 10 μl of Kapa Hotstart High Fidelity Master Mix (Kapa Technologies), and 25 ng of genomic DNA (gDNA). Thermocycling conditions were 95°C for 3 min, 98°C for 15 s, and 40°C for 30 s for 35 cycles. This cycle was followed by a last extension step at 72°C for 5 min. Equimolar amounts of barcoded amplicons from each sample were pooled and sequenced on the Ion Torrent PGM platform using the Ion PGM Sequencing 400 Kit (Thermo Scientific). We used the *split_libraries.py* script available in *Qiime* [30] and removed the sequences with an average quality score < 20 using a 50-nt sliding window and identified the barcodes used for each fecal sample allowing a maximum of two mismatches. Sequences with no barcodes and < 200 nt or > 500 nt after barcode removal were discarded. PCR primers identified at the start or end of the reads, allowing a maximum of 4 nt mismatches, were trimmed, and sequences with no identifiable primers were discarded. After primer trimming, we removed all sequences < 200 nt, and the remaining sequences were used as input for downstream analysis. Filtered sequences were clustered with 97% identity using UPARSE [31]. The seed sequence of each cluster was selected as a representative. Taxonomy was assigned by the RDP classifier [32] using a minimum confidence value of 80%. Due to unequal sequence sampling depths across samples, samples were normalized using rarefaction. Pairwise UniFrac [33] and Faith’s phylogenetic diversity [34], two metrics that compare the relative relatedness of microbial communities considering taxonomic proximity, were calculated by aligning operational taxonomic unit (OTU)-representative sequences using PyNAST [30] against the aligned greengenes core set [35] using Qiime [30] default parameters. The alignments were lane-mask filtered to construct a phylogenetic tree using FastTree [36].

### Statistical analysis

Descriptive statistics were performed using GraphPad Prism 7 (GraphPad Software, USA). The data are presented as the means ± SEM (standard error of the mean). Student’s t-test and ANOVA followed by Tukey’s multiple comparisons post hoc test were used for comparisons of 2 and 3 or more groups, respectively. A value of p ≤ 0.05 was considered significant. For microbiota statistical analysis, we used ANCOM-BC (v.1.0.1) [37] with the default parameters and a library size cutoff of 1000 reads and no structural zero detection. We assessed interactions between variables using the R limma [38] package and associations in microbial community structure with the analysis of similarities (ANOSIM) function available in the vegan R package.

## Results

### Preventive effects of oral *Bifidobacterium longum* 5^1A^ supplementation on OVA-induced allergic airway inflammation in A/J mice

In a previous work, we showed that *B. longum* 5^1A^ is an important acetate producer which confers anti-inflammatory effects on the host. Low levels of SCFAs, especially acetate, have been associated with allergic disease [21]. We measured three SCFAs, acetate, propionate, and butyrate, in the *Bifidobacterium longum* 5^1A^ culture medium [39]. Our analysis indicated that acetate was the only SCFA that could be quantified in the bacterial medium, with no measurable levels of propionate or butyrate [39]. Thus, mice were treated with probiotic and post-biotic (acetate) to investigate the mechanisms by which the probiotic was acting in the host.

**Fig. 1:**
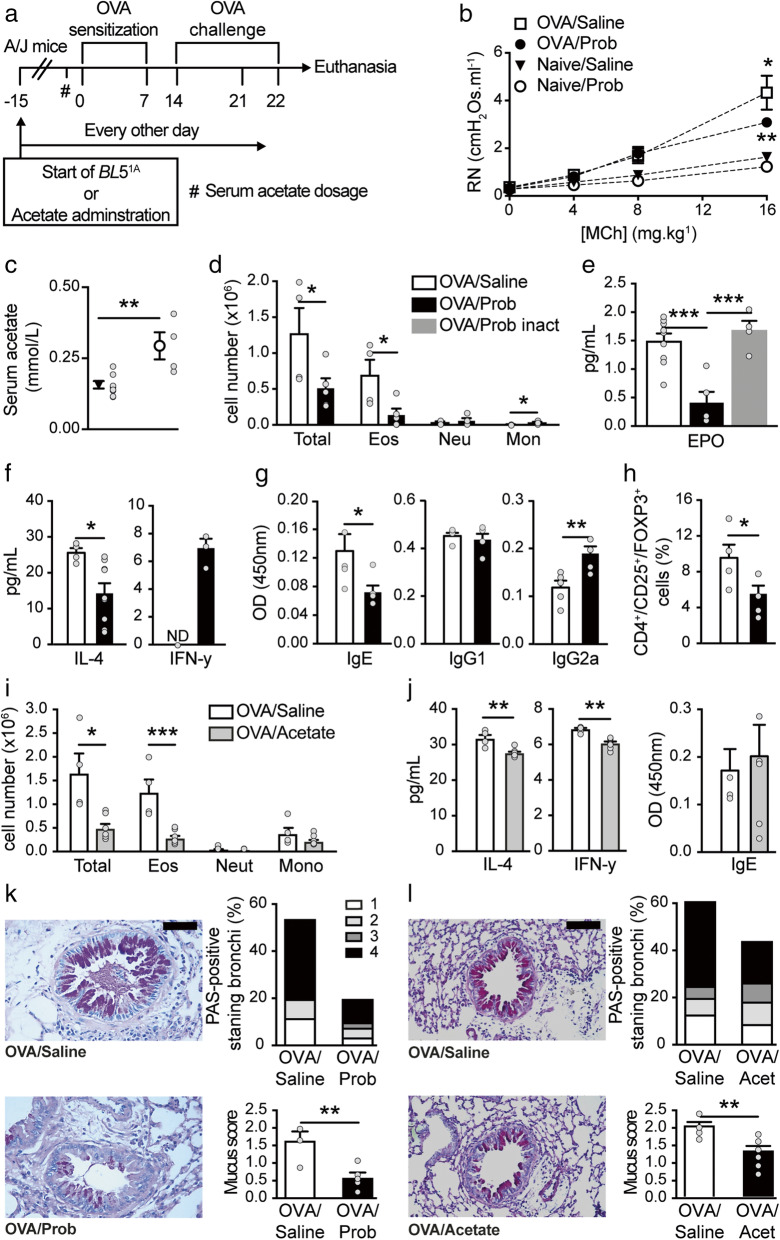
Oral acetate-producing bacteria prevent airway inflammation in A/J mice. **a** Schematic representation of the OVA-inducing airway inflammation protocol and *Bifidobacterium longum* 5^1A^ (*BL*5^1A^, Prob) or acetate administration in A/J mice. **b** Measurement of airway hyperresponsiveness (AHR) as assessed by Newtonian airway resistance (Rn) to increasing doses of methacholine in the OVA/saline, OVA/Prob, naive/saline, and naive/Prob groups (n = 4–7). **c** Levels (mmol/l) of acetate in the serum of the saline and Prob-naive groups (n = 4-8). ^#^Acetate measurement 24 h before sensitization. **d** Total and differential (Eos, eosinophils; Neut, neutrophils; Mono, mononuclear) number of cells in the bronchoalveolar lavage (BALF) of the OVA/saline and OVA/Prob groups (n = 4–5 mice per group). **e** Levels (pg/ml) of eosinophilic peroxidase (EPO) in lung tissue of the OVA/saline, OVA/Prob, and OVA/Prob-inactivated (inact) groups (n = 4–9). **f** Levels (pg/ml) of interleukin (IL)-4 and interferon (INF)-γ in the BALF of the OVA/saline and OVA/Prob groups (n = 4–6 mice per group). **g** Total amount of OVA-specific IgE, IgG1, and IgG2a in the serum of the OVA/saline and OVA/Prob groups (n = 5). **h** Percentage of positive Treg cells (CD4^+^/CD25^+^/FoxP3^+^) in the BALF of the OVA/saline and OVA/Prob groups (n = 4–6). **i** Total and differential (Eos, eosinophils; Neut, neutrophils; Mono, mononuclear) number of cells in the BALF of the OVA/saline and OVA/acetate groups (n = 4–6). **j** Levels (pg/ml) of interleukin (IL)-4 and (INF)-γ in the BALF of the OVA/saline and OVA/acetate groups (n = 4–6). **k** PAS-stained formalin-fixed histological sections of the lungs of the OVA/saline and OVA/Prob groups; scale bar represents 50 μm (×200 magnification, one representative of at least five), relative score and proportion of PAS-positive bronchi. **l** PAS-stained formalin-fixed histological sections of the lungs of the OVA/saline and OVA/acetate groups; scale bar represents 50 μm (×200 magnification, one representative of at least five), relative score and proportion of PAS-positive bronchi. The results are shown as mean ± SEM. Statistical significance was determined using Student’s t-test and ANOVA (with Tukey post-test) where appropriate (*p < 0.05, **p < 0.01, ***p < 0.001. ND, not detected). Data represent two independent experiments

Allergic airway inflammation was induced via OVA sensitization in A/J mice. Experimental groups included animals that received a *Bifidobacterium* probiotic (Prob) or sodium acetate throughout the experimental period, and controls received saline (Fig. 1A). Airway hyperreactivity (AHR) is a hallmark of asthma. Therefore, we quantified the respiratory burst of Newtonian resistance (Rn) in response to intravenous MCh 24 h after the last re-exposure to the OVA challenge. Compared to the control condition, OVA-induced lung inflammation increased AHR (Fig. 1B). A/J mice received *Bifidobacterium longum* 5^1A^ (OVA/Prob) or saline solution (OVA/Saline) via gavage beginning 15 days before the induction of OVA-mediated inflammation until the end of the protocol (Fig. 1A). Compared to the mice that received a saline solution, mice that received *Bifidobaterium longum* 5^1A^ showed decreased AHR (Fig. 1B). We measured the levels of acetate in serum using gas chromatography. Probiotic-supplemented mice showed increased acetate levels in the serum compared to non-treated mice (Fig. 1C).

We also observed increased leukocyte infiltration in BALF in the OVA group, which had a predominance of eosinophilia (Fig. 1D) and mucus overproduction (Fig. 1I). Mice that received the probiotic exhibited reduced cellular infiltration in the airways (Fig. 1D), primarily due to decreased eosinophil infiltration and increased mononuclear cells (Fig. 1D). To investigate the mechanism of probiotic modulation of lung inflammation, we inactivated the probiotic bacteria and examined whether the beneficial effect was viability-dependent. Oral administration of inactivated *Bifidobacterium longum* 5^1A^ did not reduce eosinophil infiltration (0.36 ± 0.1995) compared to the mice that received saline (0.41 ± 0.0798), and the levels of eosinophil peroxidase (EPO), which is a useful marker of lung eosinophilic activation (Fig. 1E). These results suggest that the bacteria must be alive to induce the effects. IL-4 cytokine levels were reduced in the BALF of mice supplemented with probiotics compared to the mice that received saline (Fig. 1F). IFN-γ was not detected in the OVA/saline group, but probiotic treatment induced the production of this cytokine (Fig. 1F).

OVA-induced IgE serum levels were reduced (Fig. 1G), and IgG2a serum levels were increased in the OVA/Prob group compared to the OVA/saline group (Fig. 1G). Because of the increase in mononuclear cells, we investigated the number of regulatory T (Treg) cells. Treg cells were more abundant in the lungs of mice in the OVA/saline group than the mice supplemented with the probiotic (Fig. 1H). Mucus production by goblet cells was also reduced in the animals that received the probiotic (Fig. 1K), with reduced mucus score and proportion of PAS-positive stained bronchi (Fig. 1K).

*Bifidobacterium longum* 5^1A^ produces acetate [29], and its beneficial effect was viability-dependent. Decreased acetate levels were observed in the serum of A/J mice. Therefore, we investigated whether the systemic administration of sodium acetate reduced lung inflammation. We injected sodium acetate (1 g/kg) intraperitoneally every other day in A/J mice, which reduced the influx of total cells and eosinophils (Fig. 1I) in the BALF of the experimental allergic mice. IL-4 cytokine levels were reduced in the BALF of mice supplemented with acetate compared to mice that received saline (Fig. 1J). IFN-γ was attenuated in the OVA/Acetate group compared to the OVA/saline group (Fig. 1J). OVA-induced IgE serum levels did not change with acetate supplementation (Fig. 1J). The mucus score and proportion of PAS-positive-stained bronchi were also reduced in the animals that received acetate (Fig. 1L).

In summary, our data showed that preventive supplementation with the acetate producer probiotic *B. longum* 5^1A^ clearly attenuated allergic airway inflammation and hyperresponsiveness in A/J mice.

### Oral *Bifidobacterium longum* 5^1A^ supplementation did not prevent OVA-induced allergic airway inflammation in C57BL/6 mice

**Fig. 2:**
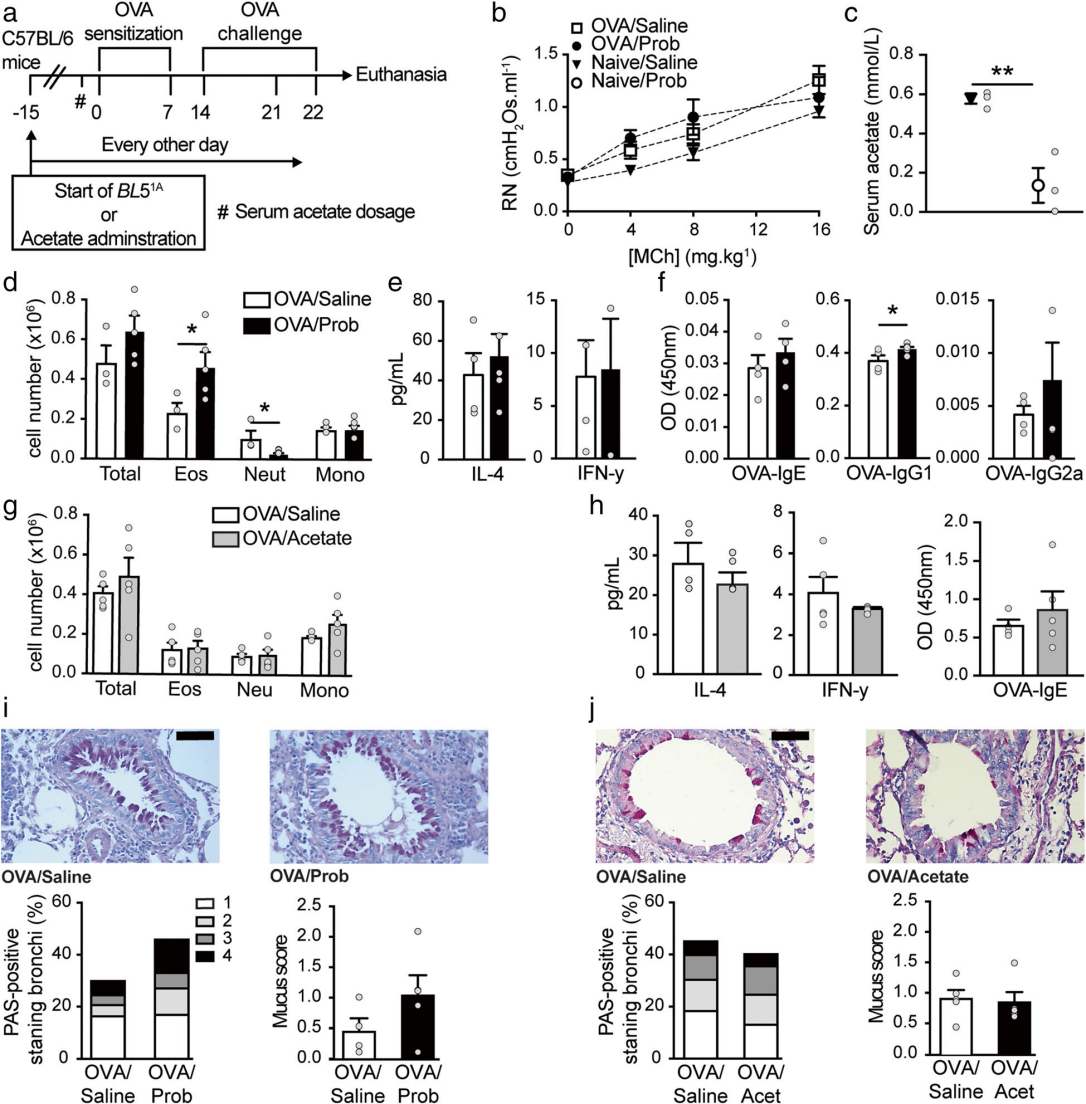
Oral acetate-producing bacteria do not prevent airway inflammation in C57BL/6 mice. **a** Schematic representation of the OVA-inducing airway inflammation protocol and *Bifidobacterium longum* 5^1A^ (*BL*5^1A^, Prob) or acetate administration in C57BL/6 (C57BL/6) mice. **b** Measurement of airway responsiveness (AHR) as assessed by Newtonian airway resistance (Rn) to increasing doses of methacholine in the OVA/saline, OVA/Prob, naive/saline, and naive/Prob groups (n = 4–5). **c** Levels of acetate (mmol/l) in the serum of the saline and Prob-naive groups (n = 3). ^#^Acetate measurement 24 h before sensitization. **d** Total and differential (Eos, eosinophils; Neut, neutrophils; Mono, mononuclear) number of cells in the bronchoalveolar lavage (BALF) of the OVA/saline and OVA/Prob groups (n = 4–5 mice per group). **e** Levels (pg/ml) of interleukin (IL)-4 and interferon (INF)-γ in the BALF of the OVA/saline and OVA/Prob groups (n = 4–5 mice per group). **f** Total amount of OVA-specific IgE, IgG1, and IgG2a in the serum of the OVA/saline and OVA/Prob groups (n = 4–5). **g** Total and differential (Eos, eosinophils; Neut, neutrophils; Mono, mononuclear) number of cells in the BALF of the OVA/saline and OVA/acetate groups (n = 5). **h** Levels (pg/ml) of interleukin (IL)-4 and interferon (INF)-γ in the BALF and OVA-specific IgE in the serum of the OVA/saline and OVA/acetate groups (n = 5). **i** PAS-stained formalin-fixed histological sections of the lungs of the OVA/saline and OVA/Prob groups; scale bar represents 50 μm (×200 magnification, one representative of at least five), relative score and proportion of PAS-positive bronchi. **j** PAS-stained formalin-fixed histological sections of the lungs of the OVA/saline and OVA/acetate groups; scale bar represents 50 μm (×200 magnification, one representative of at least five), relative score and proportion of PAS-positive bronchi. The results are shown as mean ± SEM. Statistical significance was determined using Student’s t-test and ANOVA (with Tukey post-test) where appropriate (**p < 0.01). Data represent two independent experiments. See also Figure S1

We administered the same treatment and induced allergic lung inflammation with OVA in C57BL/6 mice as described in the “Materials and methods” section (Fig. 2A). Experimental allergic C57BL/6 mice treated with *Bifidobacterium longum* 5^1A^ (OVA/Prob) did not exhibit reduced AHR compared with C57BL/6 mice in the OVA/saline group (Fig. 2B). Probiotic-supplemented C57BL/6 mice showed a decreased acetate levels in serum compared to non-treated mice (Fig. 2C). Although the probiotic treatment did not change the total cell infiltration (Fig. 2D), the inflammation was characterized by an increase in eosinophils and a decrease in neutrophils (Fig. 2D). Probiotic treatment did not change IL-4 and IFN-y cytokines levels in BALF (Fig. 2E). Probiotic treatment did not affect IgE-OVA antibody (Fig. 2F) or mucus secretion (Fig. 2I). Because probiotic supplementation did not improve eosinophilic lung inflammation in the C57BL/6 mice, we performed a complementary experiment in which the C57BL/6 mice were fed a high-fiber diet to investigate the effects of this diet on allergic inflammation (Fig. S1). Dietary fiber is associated with beneficial effects on inflammatory bowel diseases and diseases outside the intestine and may be more effective in the regulation of the immune system [40–42]. We used citrus pectin as a soluble dietary fiber that is fermented by certain species of gut bacteria, which leads to acetate production. C57BL/6 mice were given a standard diet (control diet) or a diet with a high fiber content (high-fiber diet) (Fig. S1). We exposed mice to the diet for 15 days before inducing inflammation with OVA throughout the duration and at the end of the protocol. The fiber diet did not reduce AHR (Fig. S1B), inflammatory lung parameters (Fig. S1C), IL-4 and IFN-y cytokines (Fig. S1D), IgE-OVA antibodies (Fig. S1E), or mucus secretion (Fig. S1F).

Although probiotic supplementation did not reduce allergic airway inflammation in C57BL/6 mice, we investigated whether the administration of sodium acetate modulated inflammation in this strain. Sodium acetate did not improve the total cell infiltrate in C57BL/6 mice (Fig. 1G) and did not change IL-4 and IFN-y cytokine levels (Fig. 2H), IgE-OVA antibodies (Fig. 2H), or mucus secretion (Fig. 2J) compared to OVA/saline treatment. In summary, our data showed that the preventive supplementation of the acetate producer probiotic *B. longum* 5^1A^ did not prevent allergic airway inflammation in C57BL/6 mice, which was observed in A/J mice.

### Strain-specific effects of probiotic administration on the gut microbiota

**Fig. 3:**
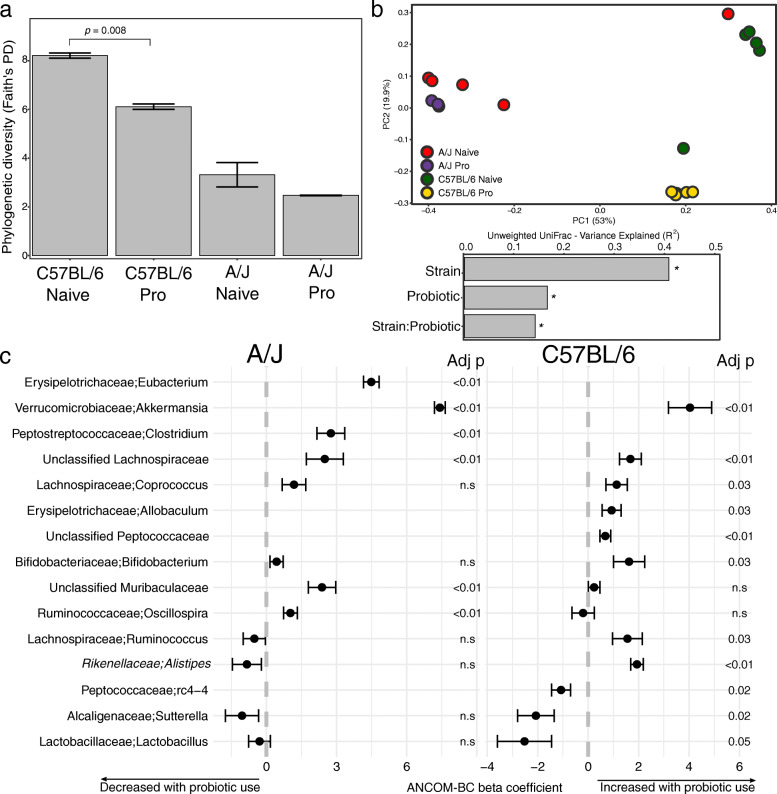
Effects of probiotic administration on murine gut microbial communities. **a** Phylogenetic diversity in mice. Error bars represent the standard error, and differences between the means were assessed using the Wilcoxon rank-sum test. **b** Microbial community variance (R^2^) explained by variables. All values were significant (p < 0.05) and were calculated using Adonis with 999 permutations on unweighted UniFrac distances. **c** Bacterial genera associated with probiotic use identified by ANCOM-BC. The genera shown have multiple hypothesis testing corrected p-values < 0.05 in one of the mouse strains. Error lines represent the standard error of the ANCOM-BC beta coefficient

We performed fecal bacterial community profiling to investigate the influence of probiotic supplementation on gut bacterial communities of A/J and C57BL/6 mice (Fig. 3). We found a greater phylogenetic diversity in C57BL/6 mice compared to A/J (p = 0.016, Wilcoxon rank-sum test), and C57BL/6 probiotic-administered mice had a significant decrease in diversity (p = 0.008, Wilcoxon rank-sum test) (Fig. 3A). Community structure differed significantly between A/J and C57BL/6 control mice (R^2^ = 0.46, p = 0.001, ADONIS). Probiotic administration was significantly associated with differences in microbial communities (R^2^ = 0.16, p = 0.001, ADONIS) and showed a combined effect with mouse strain on the overall community structure (interaction strain-probiotic R^2^ = 0.13, p = 0.002) (Fig. 3B). Probiotic administration led to an increased abundance of *Lachnospiraceae* and *Akkermansia* in both strains. The bacterial genus *Akkermansia* was not detected in either naive mouse strains, but it increased after probiotic administration, with a greater effect size in A/J mice (6.5 in A/J vs. 0.97 in C57BL/6, Cohen’s D), and showed a combined effect between mouse strain and probiotic administration (interaction strain-probiotic adjusted p-value < 0.001). C57BL/6 probiotic administered mice exhibited a significant increase in a number of genera, including *Coporoccus*, *Ruminococcus*, *Allobaculum*, and *Bifidobacterium*, and a decrease in *Lactobacillus*, *Sutterela*, and *rc4-4*. Probiotic-administered A/J mice had an increased abundance of *Clostridium*, *Eubacterium*, *Muribaculaceae*, and *Oscillospira* (Fig. 3C). Overall, these results indicate strain-specific changes in the gut microbiota after probiotic administration.

### A/J embryo implantation in C57BL/6 mothers led to a similar gut microbiota profile and allergic airway inflammation to C57BL/6 offspring

**Fig. 4:**
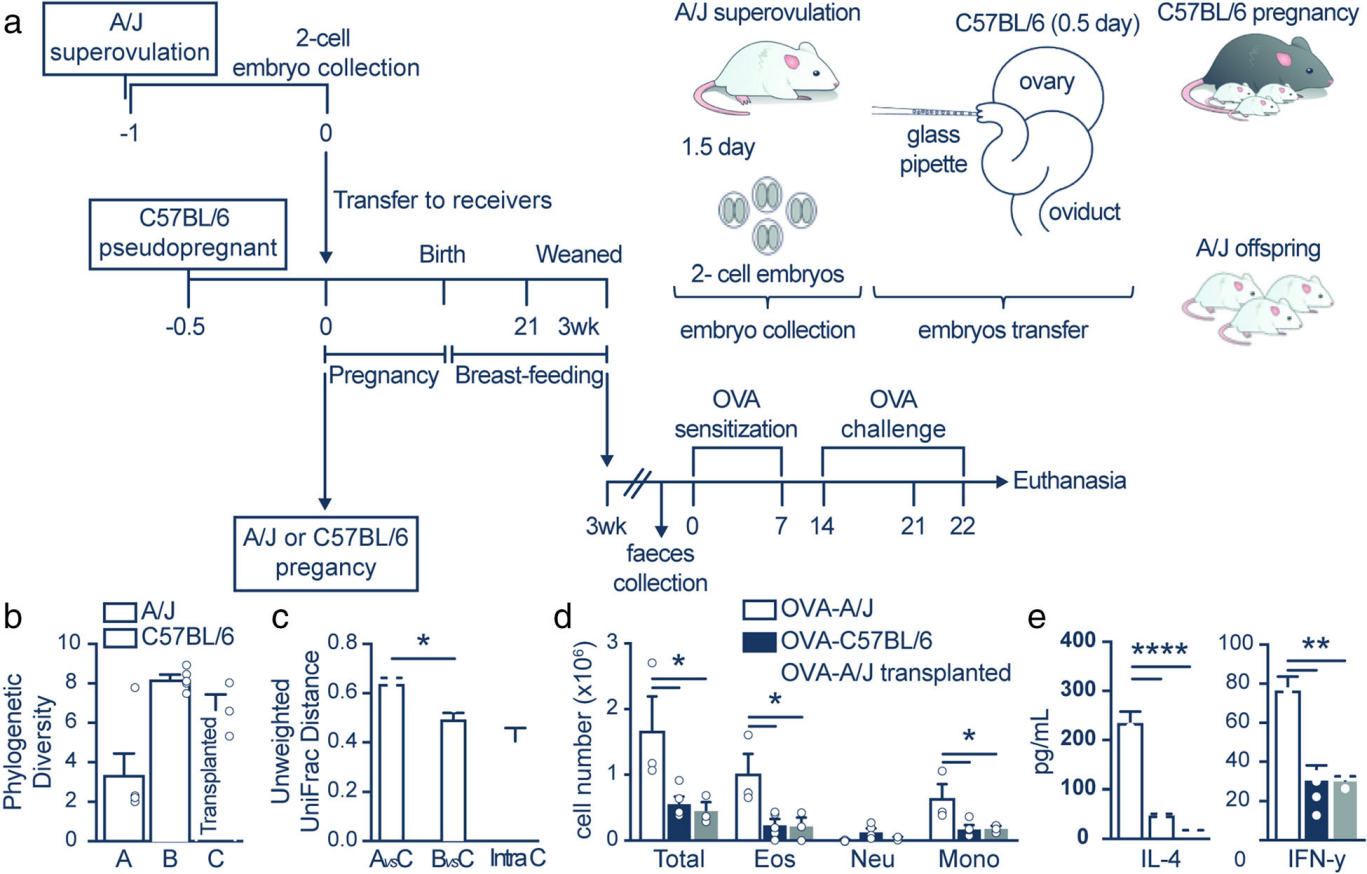
A/J embryo implantation in C57BL/6 mothers resulted in experimental allergic airway inflammation similar to C57BL/6 offspring. **a** Schematic representation of the embryo transfer and OVA-inducing airway inflammation protocol. **b** Phylogenetic diversity in mice. Error bars represent the standard error, and differences between the means were assessed using the Wilcoxon rank-sum test. **c** Microbial community variance (R^2^) explained by variables. All values were calculated using Adonis with 999 permutations on unweighted UniFrac distances. **d** Total and differential (Eos, eosinophils; Neut, neutrophils; Mono, mononuclear) number of cells in the bronchoalveolar lavage (BALF) of the A/J, C57BL/6, and A/J transplanted OVA groups (n = 3–5). **e** Levels of (pg/ml) of interleukin (IL)-4 and interferon (INF)-γ in the BALF of the A/J, C57BL/6, and A/J transplanted OVA groups (n = 3–5). The results are shown as mean ± SEM. ANOVA test (with Tukey post-test) was used. *p < 0.05, **p < 0.01, ****p < 0.0001

To better understand the relevance of microbial composition colonization to the development of allergic diseases, we implanted a female C57BL/6 mouse with A/J embryos to naturally modulate the microbial composition of A/J mice, and the fecal gut microbiome of the offspring was profiled 6 weeks after birth (Fig. 4A). A/J-transplanted mice exhibited increased phylogenetic diversity compared to A/J mice, but this diversity was lower than the C57BL/6 mice (Fig. 4B). We found that the gut microbial communities of A/J-transplanted mice were more similar to C57BL/6 mice than their genetically similar counterparts (*p* = 0.002, Wilcoxon rank-sum test) (Fig. 4C).

We assessed whether allergic lung inflammation differed due to the differences in the environment by inducing allergic lung inflammation with OVA in the transplanted A/J mice and A/J and C57BL/6 mice (Fig. 4A). Compared to non-transplanted A/J mice, transplanted A/J mice exhibited reduced cellular infiltration in the airways (Fig. 4D), primarily due to decreased eosinophilic infiltration and mononuclear cells, similar to C57BL/6 mice. Similar levels of IL-4 and IFN-γ cytokines were observed in the BALF of C57BL/6 mice and transplanted A/J mice, and these cytokines were increased in A/J mice compared to transplanted A/J mice and C57BL/6 mice (Fig. 4E). These observations suggest that the environment significantly influenced the gut microbiota, which may lead to the differences in the allergic airway inflammation phenotype.

## Discussion

Genetic predisposition to complex diseases partially manifests as an inclination to aberrant patterns of microbial colonization, which contributes to disease processes [43]. Allergic inflammation is related to gut microbiota dysbiosis. The gut microbiota is relevant to allergic airway inflammation, and different strategies were used to modulate this microbiota, including dietary supplementation with probiotics [44]. However, few studies demonstrated that the probiotic effects on allergic disease may be related to host genetics and the microbiota composition. Our group recently showed that preventive oral *Bifidobacterium adolescentis* ATCC 15703 treatment attenuated the major characteristics of allergic asthma and eosinophilic airway influx in BALB/c mice, but not in C57BL/6 mice, but the gut microbiota was not evaluated in that study [20]. *Bifidobacterium* is a potential protective agent against allergic disease, and we selected a *Bifidobacterium* probiotic for investigation [45]. *Bifidobacterium longum* 5^1A^, which was isolated from the stool of a healthy child, exerted probiotic effects on the host [22, 39, 46–49]. Notably, sequencing of the *Bifidobacterium longum* 5^1A^ genome revealed two unique genes, Ll51A 1408 and Ll51A 1405, which correspond to a carbohydrate kinase that participates in the phosphoketolase pathway that is unique to *Bifidobacterium* and may be involved in acetate production (unpublished data). We observed that the administration of the same probiotic, *Bifidobacterium longum* 5^1A^, to two mouse strains induced different changes in the microbiota composition and acetate levels in the serum and had adverse effects on lung inflammation. *Bifidobacterium longum* 5^1A^ administration in A/J mice decreased airway eosinophilic infiltration and reduced bronchial responsiveness and OVA-specific IgE production. These effects were dependent on live bacteria because airway inflammation was not changed when the bacteria were inactivated. Notably, supplementation with *Bifidobacterium longum* 5^1A^ in C57BL/6 mice did not prevent exacerbated eosinophilic airway inflammation. The prevention of eosinophilic inflammation in A/J mice by probiotics was associated with increased levels of acetate in the plasma. We demonstrated that *Bifidobacterium longum* 5^1A^ was a producer of acetate [39], and its beneficial probiotic effect in A/J mice was at least partially due to acetate production, because inactivated bacteria did not affect airway inflammation. Acetate is an SCFA known that acts via two main mechanisms: signaling via G-protein-coupled receptors (GPCRs), such as GPR43, GPR41, and GPR109A, and inhibition of histone deacetylases (HDACs), which affects gene transcription [42]. Both of these mechanisms inhibit nuclear factor kappa B (NF-кB) activity, which leads to anti-inflammatory effects, including decreased neutrophil migration, decreased proinflammatory cytokine production, and increased Treg cells [50]. We found that probiotic treatment of allergic A/J mice did not increase Treg cell levels, which may be because airway eosinophilia was already resolving 24 h after the challenge.

We injected acetate intraperitoneally in A/J and C57BL/6 mice to examine the systemic acetate effect. The intraperitoneal administration of acetate in A/J mice decreased allergic airway inflammation but did not impact C57BL/6 mice. The effect of acetate in drinking water on airway inflammation was demonstrated in C57BL/6 mice [12, 42], but the effect of acetate i.p. administration on allergic airway inflammation is poorly described.

The different outcomes of probiotic administration in this paper may be explained by the differences in naive gut microbiota between mouse strains. A/J mice showed a different microbiota than C57BL/6 mice, especially in diversity. Mice with a resistant microbiota have a more robust microbial structure and exhibit less susceptibility to changes induced by probiotics than permissive microbiota [51]. The composition of the gut microbiota in C57BL/6 mice had greater diversity than A/J mice. Therefore, probiotic administration modulates microbiota diversity differently in various mouse strains. Notably, probiotic treatment reduced diversity in C57BL/6 mice but not in AJ mice, and the reduced diversity in C57BL/6 mice was associated with decreased levels of acetate in the serum. A/J mice also had more pronounced Th2 inflammation than C57BL/6 mice. Consistent with these findings, previous studies reported an association between lower microbiota diversity during the first month of life and asthma development at 7 years of age [52–54]. High levels of four genera, *Lachnospira*, *Veillonella*, *Faecalibacterium*, and *Rothia*, in the stool at 3 months of age were associated with the development of early asthma or allergy symptoms [55]. Probiotic treatment increased some bacteria in different manners in A/J mice and C57BL/6 mice in the present study. *Akkermansia* was increased in A/J mice, and C57BL/6 mice exhibited increased *Alistipes* and decreased *Lactobacillus* after probiotic treatment. *Akkermansia*-like spp. belong to the Verrucomicrobia family and are present in approximately 3% of healthy individuals. This family strengthened the gut barrier function in mice [56]. One study showed that feeding mice a fiber-free diet caused damage to the mucus barrier because *Akkermansia muciniphila* and other bacteria switched their metabolism from fiber degradation to mucus glycan degradation. Notably, *A. muciniphila* also produced acetate and propionate [57, 58]. *A. muciniphila* was decreased in patients with ulcerative colitis and Crohn’s disease [59] and exerted anti-inflammatory effects via its metabolites, which control genes that regulate bowel function, especially in host intestinal epithelial cells. The abundance of *A. muciniphila* was also inversely associated with body fat mass and glucose intolerance in mice [60]. Reduced *A. muciniphila* and *Faecalibacterium prausnitzii* levels in the gut microbiota were associated with allergic asthma in children [61]. Higher levels of *Alistipes* were found in C57BL/6 mice after probiotic treatment. Published findings on the associations between *Alistipes* and inflammation and allergic disease are rare. However, a previous study showed that genetic iTreg cell-deficient mice (background C57BL/6) spontaneously developed Th2-type pathologies in the local mucosa, such as the gastrointestinal tract and lungs. This deficiency of iTreg cells in the lungs induced characteristics of allergic inflammation and asthma. The gut microbiota of the iTreg-deficient mice was also altered, with an increase in the phylum TM7 and the genus *Alistipes* and a decrease in the ratio of Firmicutes to Bacteroidetes [62]. High levels of *Alistipes* were also present in tumor-bearing mice [63] and colorectal carcinogenesis [64]. Another finding of our study was that C57BL/6 mice had more *Lactobacillus* in the gut than A/J mice, but probiotic treatment decreased the levels of these bacteria. *Lactobacillus* increases Treg cells, which is important for allergic resolution [21, 65]. *Lactobacillus* supplementation temporarily modified delayed gut microbiota development in high-risk asthmatic infants [66].

To naturally change the gut microbiota composition and diversity in A/J mice, we implanted a female C57BL/6 mouse with A/J embryos. We observed that genetic background was not sufficient to determine the intensity of allergic airway inflammation in inbred mouse strains because transplanted A/J mice developed allergic airway inflammation similar to C57BL/6 mice and not like non-transplanted A/J mice. Transplanted A/J mice exhibited a gut microbiota composition and diversity that was similar to C57BL/6 mice, a gestational surrogate strain. We used the embryo transfer method to transfer microbiota because the pups generated are exposed to the maternal microbiota during gestation and immediately at birth and acquire microbial communities via natural means. C57BL/6 to C57BL/6 implantation was not performed because previous studies showed that shifts in the microbiota and immune parameters occurred in offspring that were embryo-transferred to surrogate dams of a different genotype but not the same genotype [67, 68]. Taken together, these results suggest that the gut microbiota composition, especially gut microbiota diversity in early life, has a relevant effect on experimental allergic airway inflammation in inbred mouse strains and overcomes genetic factors related to allergic inflammation. The embryo transfer experiment showed that early life events were important in microbiota formation. Many studies indicated that early life events, such as delivery and breastfeeding, played a fundamental role in microbiota formation and influenced health and disease [43, 69]. The fetal gut was long assumed to be sterile, with colonization occurring only at delivery [13]. Although controversial, the idea that microbiota colonization starts in utero was recently described with the apparent identification of low-abundance bacteria in fetal membranes, amniotic fluid, and placental tissue [70–73]. The postpartum period, including breastmilk feeding, is very relevant to microbiota formation [74]. One study used a large murine intercross model in which the genetic background was systematically evaluated, and environmental factors were controlled and showed that host genetic control shaped individual microbiome diversity in mammals [43]. Notably, another study implanted a female BDF1 mouse with three Agouti (Ag) and three C57BL/6 embryos and analyzed fecal samples from the offspring and mother at weekly intervals following weaning (3 weeks of age) until 10 weeks. This study found dendrogram concordance in excess of 93% for gut microbiota [75].

Person-specific variations in microbiome composition and function contribute to the variability in glycemic responses to a variety of foods [76] and synthetic food supplements [77]. Some studies showed differences in disease outcomes between mice of the same strain but from different mouse facilities or vendors. Two independent studies recently demonstrated that gut microbiota reconciled different responses to immune checkpoint inhibitors in mouse models of melanoma, and tumor growth varied depending on whether the mice were obtained from The Jackson Laboratory or Taconic vendors. These mice had the same genetic background (C57BL/6) but distinct microbial compositions. Tumors grew slower and responded more robustly to anti-PD-L1 immunotherapy in JAX mice than Taconic mice. Fecal microbiota transplantation from JAX donors to Taconic recipients enhanced the anti-PD-L1 tumor efficacy [78]. The authors identified *Bifidobacterium* as a crucial agent for mediating anti-PD-L1 efficacy by altering dendritic cell activity that enhanced CD8-positive T cell responses to destroy tumors [78]. A/J and C57BL/6 mice in other mouse facilities may have different microbiota and respond differently to *Bifidobacterium longum* 5^1A^, acetate, and diet.

## Conclusion

**Fig. 5:**
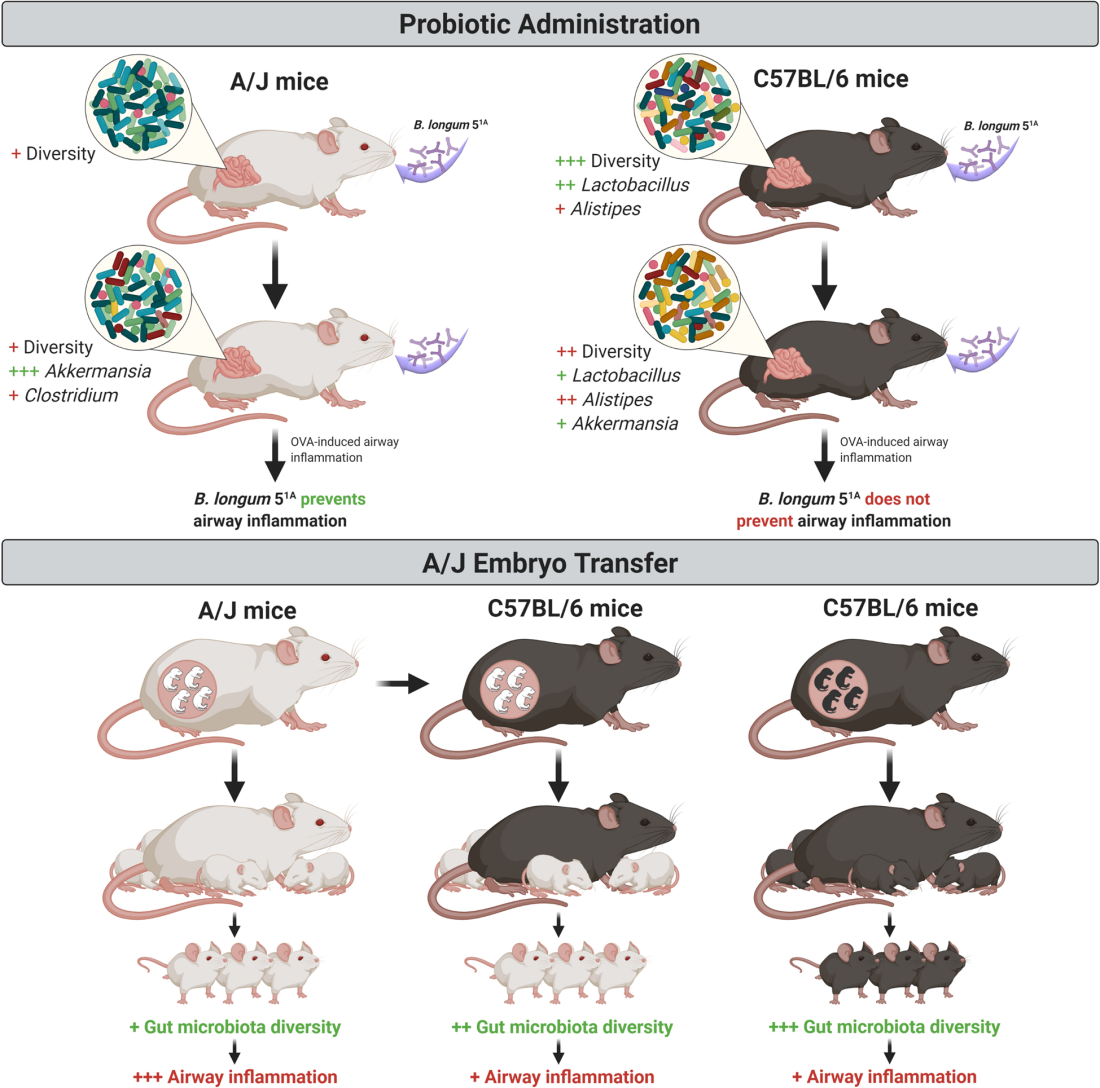
Schematic representation of the effect of *Bifidobacterium longum* 5^1A^ administration and embryo transfer on experimental allergic airway disease in A/J and C57BL/6

Our data showed that (a) the use of the same probiotic induced different changes in gut microbiota, which correlated with the host gut microbiota, but that these changes may not always have positive implications for health, and adverse effects may occur, and (b) the microbiota composition was very relevant to the allergic airway inflammation phenotype (Fig. 5). Therefore, the indiscriminate use of probiotics should be reconsidered because the effects of these products are dependent on host-related parameters, such as the resident gut microbiota.

## Supplementary Information

**Additional file 1:.** Figure S1. High-fiber diet do not prevent airway inflammation in B6 mice. (A) Schematic representation of the OVA-inducing airway inflammation protocol and High-fiber diet in C57BL/6 mice. (B) Measurement of airway responsiveness (AHR) as assessed by Newtonian airway resistance (Rn) to increasing doses of methacholine in Control diet (CD) and High-fiber diet (HFD) of naive and OVA groups (n=4-5). (C) Total and differential (Eos: eosinophils; Neut: neutrophils; Mono: mononuclear) number of cells in the bronchoalveolar lavage (BALF) of OVA/Control Diet and OVA/High-fiber Diet groups (n=3-5 mice per group); (D) Concentrations (pg/ml) of interleukin (IL)-4 and interferon (INF)-γ in the BALF of OVA/Control Diet and OVA/High-fiber Diet groups (n=4-5); (E) Total amount of OVA-specific IgE in the serum of OVA/Control Diet and OVA/High-fiber Diet groups (n=5); (F) Representative PAS-stained bronchial structure of OVA/Control Diet and OVA/High-fiber Diet groups, scale bar represents 50 μm (200x magnification, one representative of at least five). Results are shown as mean ± SEM. Statistical significance was determined using Student’s t-test and ANOVA (with Tukey post-test) where appropriated. Panels provide compiled data of two independent experiments. Figure S2. Heatmap showing log transformed relative abundances of differentially abundant bacterial genera found between naive and probiotic administered mice. Figure S3. Principal component analysis (PCoA) on UnWeighted UniFrac distances.

## Data Availability

Supporting information is available online in association with this paper. Sequences were uploaded to the NCBI’s Sequence under accession number PRJNA669826 (fecal samples).
