# Correction to: A probiotic has differential effects on allergic airway inflammation in A/J and C57BL/6 mice and is correlated with the gut microbiome

**DOI:** 10.1186/s40168-021-01116-8

**Published:** 2021-07-14

**Authors:** Mateus B. Casaro, Andrew M. Thomas, Eduardo Mendes, Claudio Fukumori, Willian R. Ribeiro, Fernando A. Oliveira, Amanda R. Crisma, Gilson M. Murata, Bruna Bizzarro, Anderson Sá-Nunes, Joao C. Setubal, Marcia P. A. Mayer, Flaviano S. Martins, Angélica T. Vieira, Ana T. F. B. Antiorio, Wothan Tavares-de-Lima, Niels O. S. Camara, Rui Curi, Emmanuel Dias-Neto, Caroline M. Ferreira

**Affiliations:** 1 Department of Pharmaceutics Sciences, Institute of Environmental, Chemistry and Pharmaceutical Sciences, Universidade Federal de São Paulo, R. São Nicolau, 210, Diadema, SP 09913-03 Brazil; 2 Department CIBIO, University of Trento, Trento, Italy; 3 Medical Genomics Laboratory, CIPE/A.C. Camargo Cancer Center, São Paulo, Brazil; 4 Department of Biochemistry, Institute of Chemistry, Universidade de São Paulo, São Paulo, Brazil; 5 Center for Mathematics, Computing and Cognition (CMCC), Federal University of ABC – UFABC, São Bernardo do Campo, SP Brazil; 6 Department of Clinical Analyses, Universidade Federal do Paraná, Curitiba, Brazil; 7 Department of Medical Clinic, Faculty of Medicine, University of São Paulo, São Paulo, 01246-903 Brazil; 8 Department of Immunology, Institute of Biomedical Sciences, Universidade de São Paulo, São Paulo, Brazil; 9 Department of Microbiology, Institute of Biomedical Sciences, University of São Paulo, São Paulo, SP Brazil; 10 Department of Microbiology, Institute of Biological Sciences, Federal Universidade de Minas Gerais, Belo Horizonte, Brazil; 11 Department of Biochemistry and Immunology, Biological Science Institute, Federal University of Minas Gerais, Belo Horizonte, Brazil; 12 Department of Pathology, School of Veterinary Medicine and Animal Science, Universidade de São Paulo, São Paulo, Brazil; 13 Department of Pharmacology, Institute of Biomedical Sciences I, Universidade de São Paulo, São Paulo, Brazil; 14 Interdisciplinary Post-Graduate Program in Health Sciences, Cruzeiro do Sul University, São Paulo, Brazil; 15 Laboratory of Neurosciences (LIM-27), Institute of Psychiatry, Medical School, Universidade de São Paulo, São Paulo, Brazil

**Correction to: Microbiome 9, 134 (2021)**

**https://doi.org/10.1186/s40168-021-01081-2**

Following the publication of the original article [1], the author reported that the bars in panels b, c and e of Fig. 4 are blank. The correct Fig. 4 is provided below.

**Fig. 4:**
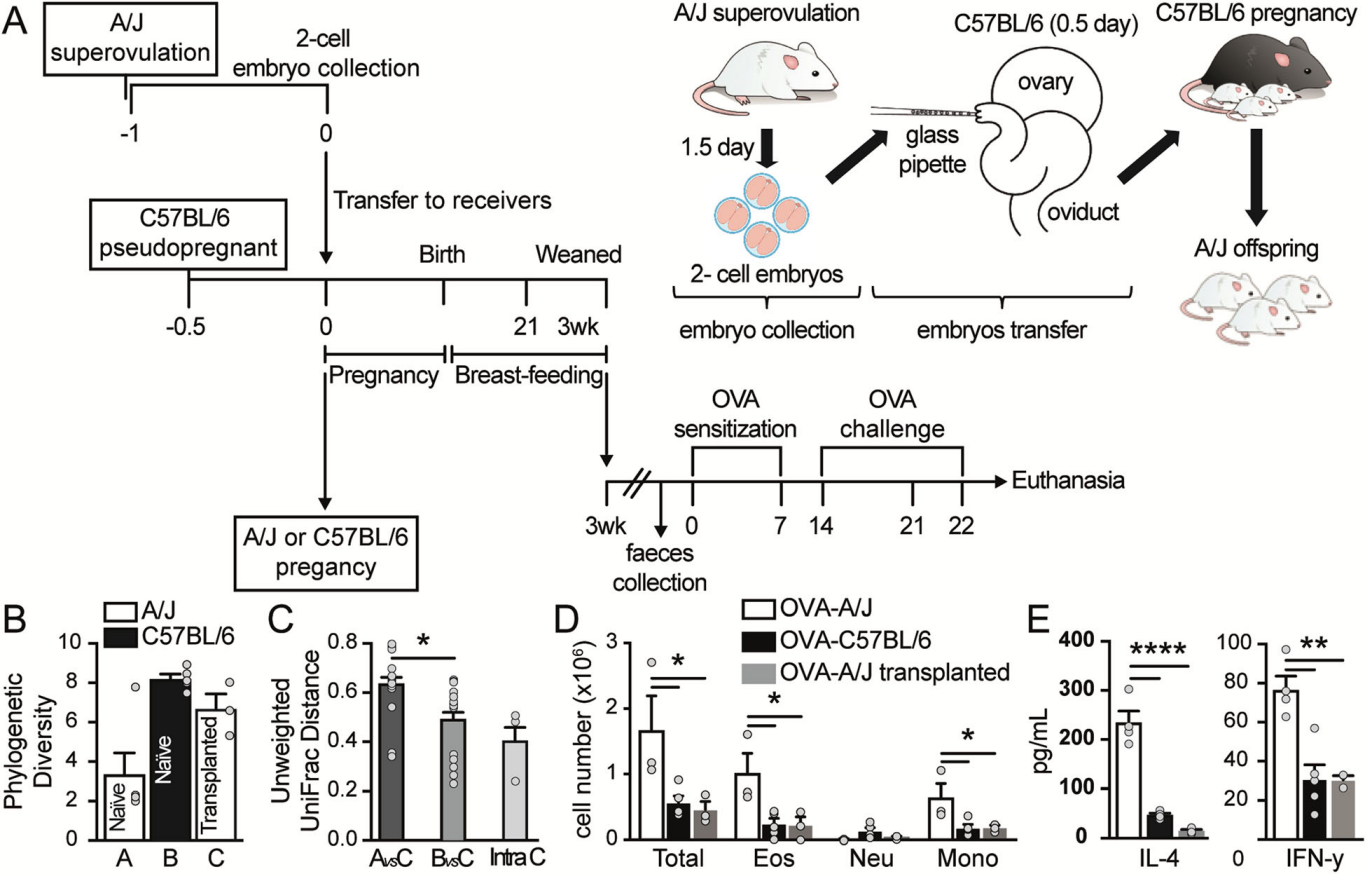
A/J embryo implantation in C57BL/6 mothers resulted in experimental allergic airway inflammation similar to C57BL/6 offspring. **a** Schematic representation of the embryo transfer and OVA-inducing airway inflammation protocol. **b** Phylogenetic diversity in mice. Error bars represent the standard error, and differences between the means were assessed using the Wilcoxon rank-sum test. **c** Microbial community variance (R^2^) explained by variables. All values were calculated using Adonis with 999 permutations on unweighted UniFrac distances. **d** Total and differential (Eos, eosinophils; Neut, neutrophils; Mono, mononuclear) number of cells in the bronchoalveolar lavage (BALF) of the A/J, C57BL/6, and A/J transplanted OVA groups (*n* = 3–5). **e** Levels of (pg/ml) of interleukin (IL)-4 and interferon (INF)-γ in the BALF of the A/J, C57BL/6, and A/J transplanted OVA groups (*n* = 3–5). The results are shown as mean ± SEM. ANOVA test (with Tukey post-test) was used. *p < 0.05, **p < 0.01, ****p < 0.0001

The original article has been updated.
