# Supplementary material for: A probiotic has differential effects on allergic airway inflammation in A/J and C57BL/6 mice and is correlated with the gut microbiome
Source: Microbiome. 2021 Jun 10;9:134. doi: 10.1186/s40168-021-01081-2 (PMC8194189; doi:10.1186/s40168-021-01081-2)
Supplement: Supplementary file 2 — Additional file 1:. Figure S1. High-fiber diet do not prevent airway inflammation in B6 mice. (A) Schematic representation of the OVA-inducing airway inflammation protocol and High-fiber diet in C57BL/6 mice. (B) Measurement of airway responsiveness (AHR) as assessed by Newtonian airway resistance (Rn) to increasing doses of methacholine in Control diet (CD) and High-fiber diet (HFD) of naive and OVA groups (n=4-5). (C) Total and differential (Eos: eosinophils; Neut: neutrophils; Mono: mononuclear) number of cells in the bronchoalveolar lavage (BALF) of OVA/Control Diet and OVA/High-fiber Diet groups (n=3-5 mice per group); (D) Concentrations (pg/ml) of interleukin (IL)-4 and interferon (INF)-γ in the BALF of OVA/Control Diet and OVA/High-fiber Diet groups (n=4-5); (E) Total amount of OVA-specific IgE in the serum of OVA/Control Diet and OVA/High-fiber Diet groups (n=5); (F) Representative PAS-stained bronchial structure of OVA/Control Diet and OVA/High-fiber Diet groups, scale bar represents 50 μm (200x magnification, one representative of at least five). Results are shown as mean ± SEM. Statistical significance was determined using Student’s t-test and ANOVA (with Tukey post-test) where appropriated. Panels provide compiled data of two independent experiments. Figure S2. Heatmap showing log transformed relative abundances of differentially abundant bacterial genera found between naive and probiotic administered mice. Figure S3. Principal component analysis (PCoA) on UnWeighted UniFrac distances. [file 40168_2021_1081_MOESM1_ESM.docx]

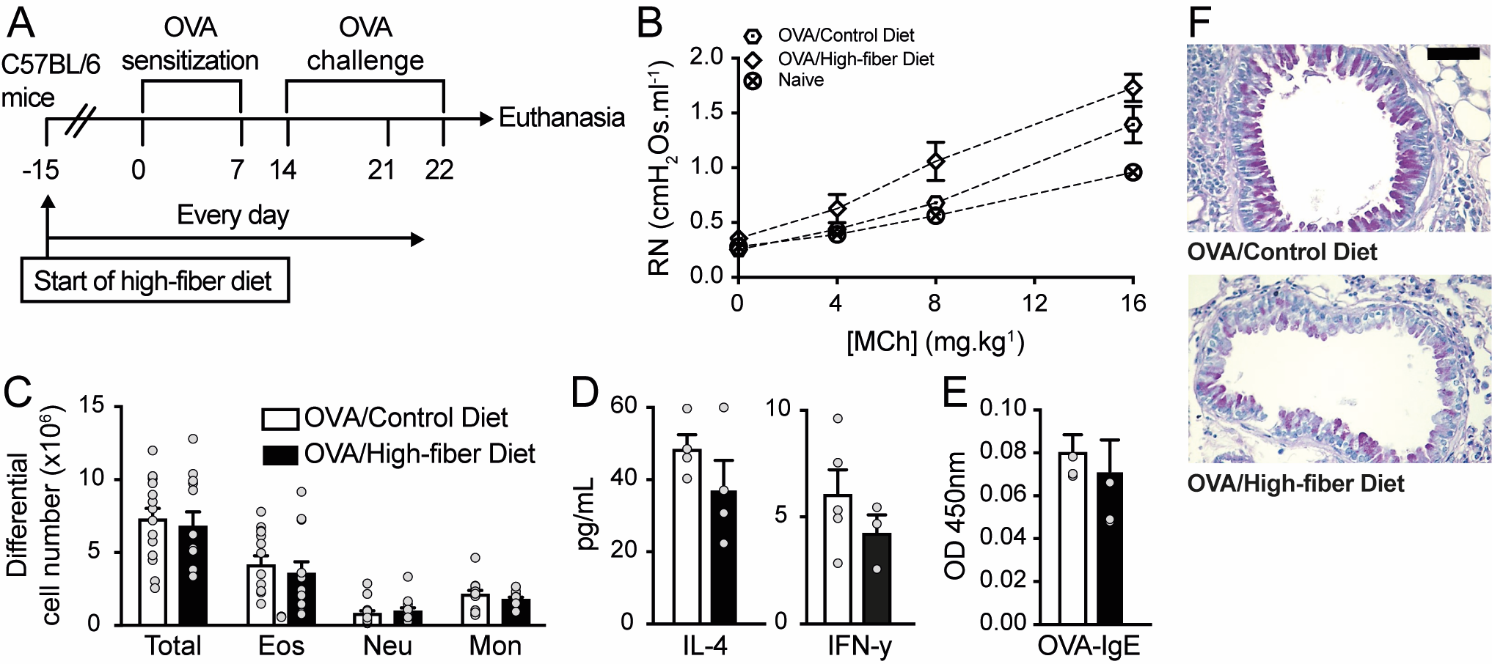


**Figure S1: High-fiber diet do not prevent airway inflammation in B6 mice**

(A) Schematic representation of the OVA-inducing airway inflammation protocol and High-fiber diet in C57BL/6 mice. (B) Measurement of airway responsiveness (AHR) as assessed by Newtonian airway resistance (Rn) to increasing doses of methacholine in Control diet (CD) and High-fiber diet (HFD) of naïve and OVA groups (n=4-5). (C) Total and differential (Eos: eosinophils; Neut: neutrophils; Mono: mononuclear) number of cells in the bronchoalveolar lavage (BALF) of OVA/Control Diet and OVA/High-fiber Diet groups (n=3-5 mice per group); (D) Concentrations (pg/ml) of interleukin (IL)-4 and interferon (INF)-γ in the BALF of OVA/Control Diet and OVA/High-fiber Diet groups (n=4-5); (E) Total amount of OVA-specific IgE in the serum of OVA/Control Diet and OVA/High-fiber Diet groups (n=5); (F) Representative PAS-stained bronchial structure of OVA/Control Diet and OVA/High-fiber Diet groups, scale bar represents 50 μm (200x magnification, one representative of at least five). Results are shown as mean ± SEM. Statistical significance was determined using Student’s t-test and ANOVA (with Tukey post-test) where appropriated. Panels provide compiled data of two independent experiments.


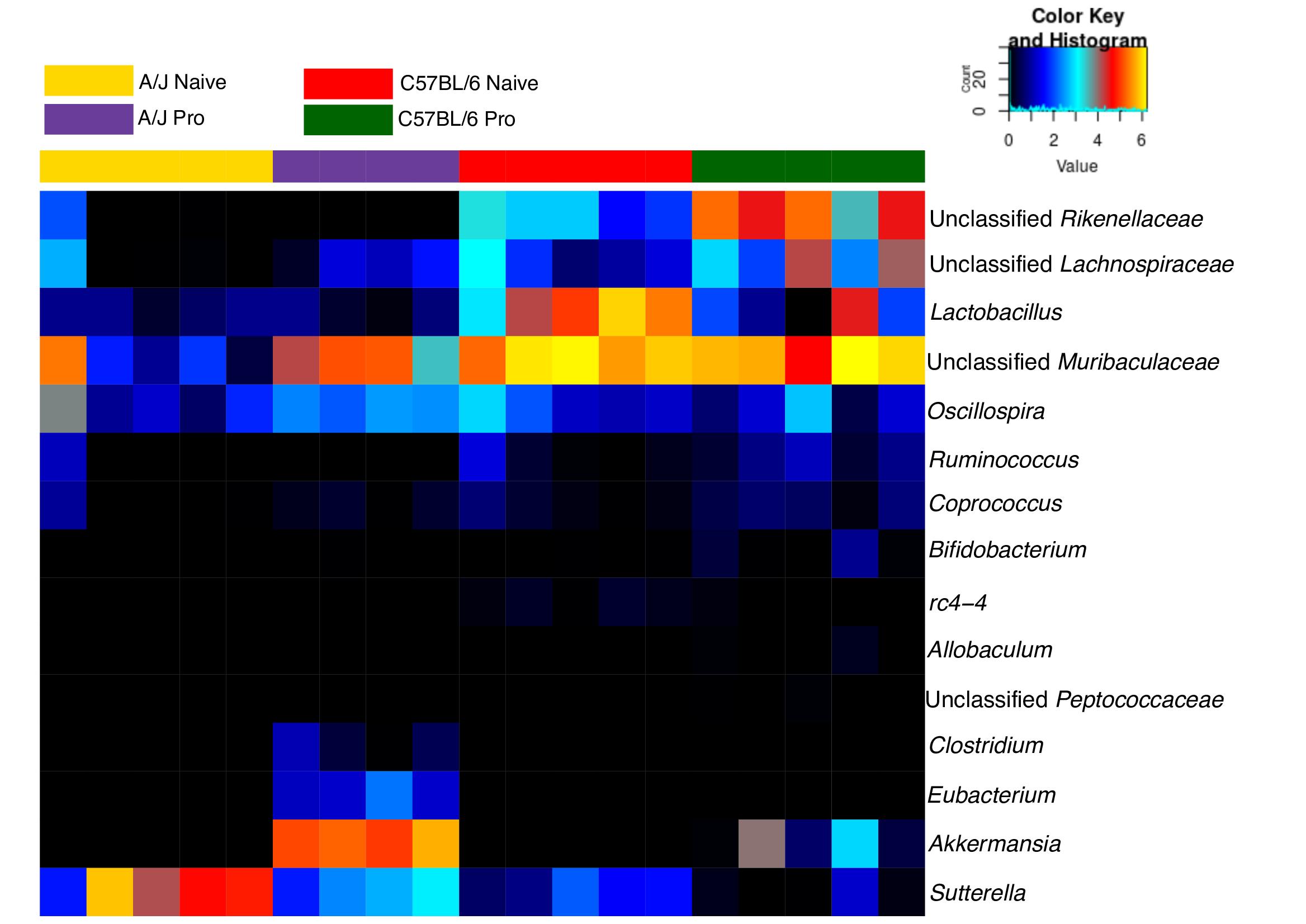


**Figure S2: Heatmap showing log transformed relative abundances of differentially abundant bacterial genera found between naïve and probiotic administered mice.**


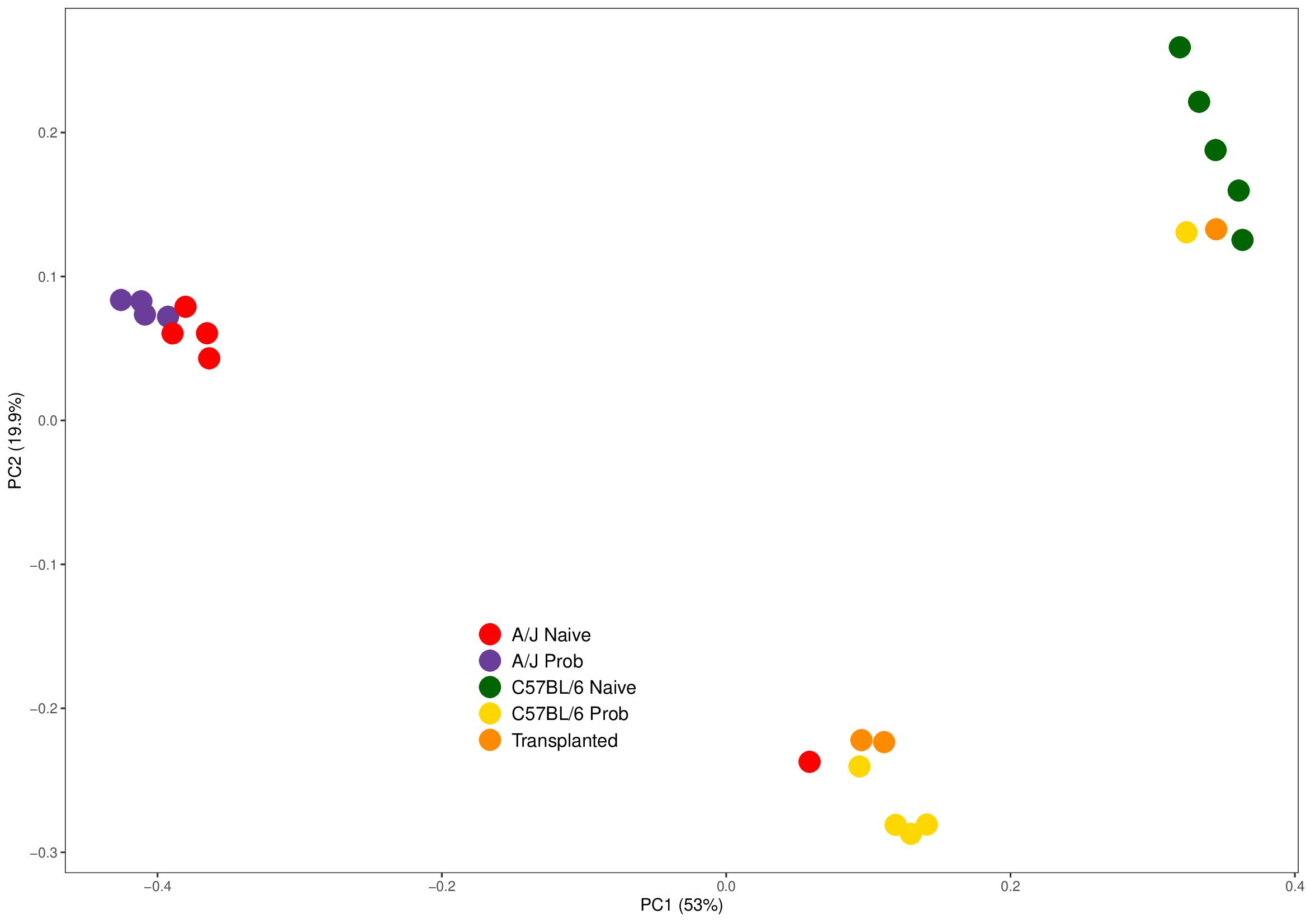


**Figure S3: Principal component analysis (PCoA) on UnWeighted UniFrac distances.**
